# ERK2 and JNK1 contribute to TNF-α-induced IL-8 expression in synovial fibroblasts

**DOI:** 10.1371/journal.pone.0182923

**Published:** 2017-08-14

**Authors:** Shinichi Namba, Rei Nakano, Taku Kitanaka, Nanako Kitanaka, Tomohiro Nakayama, Hiroshi Sugiya

**Affiliations:** 1 Laboratory of Veterinary Biochemistry, Department of Veterinary Medicine, College of Bioresource Sciences, Nihon University, Kameino, Fujisawa, Kanagawa, Japan; 2 Laboratory of Veterinary Radiology, Department of Veterinary Medicine, College of Bioresource Sciences, Nihon University, Kameino, Fujisawa, Kanagawa, Japan; Chang Gung University, TAIWAN

## Abstract

Tumor necrosis factor α (TNF-α) induces the expression and secretion of interleukin 8 (IL-8), which contributes to synovitis in rheumatoid arthritis (RA). To elucidate the mechanism of the onset of RA, we used synovial fibroblasts without autoimmune inflammatory diseases and investigated MAPK signaling pathways in TNF-α-induced IL-8 expression. Synovial fibroblasts isolated from healthy dogs were characterized by flow cytometry, which were positive for the fibroblast markers CD29, CD44, and CD90 but negative for the hematopoietic cell markers CD14, CD34, CD45, and HLA-DR. TNF-α stimulated the secretion and mRNA expression of IL-8 in a time- and dose-dependent manner. ERK and JNK inhibitors attenuated TNF-α-induced IL-8 expression and secretion. TNF-α induced the phosphorylation of ERK1/2 and JNK1/2. TNF-α-induced IL-8 expression was attenuated both in ERK2- and JNK1-knockdown cells. TNF-α-induced ERK1/2 or JNK1/2 was observed in ERK2- or JNK1-knockdown cells, respectively, showing that there is no crosstalk between ERK2 and JNK1 pathways. These observations indicate that the individual activation of ERK2 and JNK1 pathways contributes to TNF-α-induced IL-8 expression in synovial fibroblasts, which appears to be involved in the progress in RA.

## Introduction

Rheumatoid arthritis (RA) is a chronic, progressive, and inflammatory autoimmune disease characterized by a failure of immune regulation in the joint synovial membrane, causing severe damage and destruction of cartilage and bone [1, 2]. Synovitis (inflammation of the synovial membrane) is recognized as a hallmark of RA. Pro-inflammatory cytokines, such as tumor necrosis factor α (TNF-α) and interleukin 1β, play a crucial role in the generation of synovitis and progressive joint destruction [3, 4].

Interleukin 8 (IL-8) is an inflammatory chemokine produced under conditions of inflammatory stimulation and mediates chemotaxis of neutrophils and angiogenesis [5, 6]. IL-8 has been detected in synovial fluids and serum of human RA patients [7–11] and in synovial fluid of knee joints of dogs with RA [12, 13]. IL-8 has also been reported to be involved in causing pain in the joints with RA [14]. Therefore, IL-8 has been considered to be the most important inflammatory chemokine associated with arthritis [15, 16]. In RA synovial fibroblasts, as well as in monocytes and chondrocytes, the pro-inflammatory cytokine TNF-α has been demonstrated to induce IL-8 mRNA expression and protein secretion [17–23], indicating that synovial fibroblasts stimulated with pro-inflammatory cytokines contribute to the production and secretion of IL-8 in inflamed joint diseases, such as RA [24–26].

Mitogen-activated protein kinases (MAPKs) play a central role in signaling pathways in synovial fibroblasts stimulated with TNF-α. Extracellular signal-regulated kinase (ERK), c-Jun N-terminal kinase (JNK), and p38 are primary mitogen activated protein kinases (MAPKs). Expression of these MAPKs, as well as of their phosphorylated active forms, was detected in synovial tissues and synovial fibroblasts of RA patients [27]. In RA synovial fibroblasts, MAPKs have been demonstrated to be involved in the TNF-α-induced expression of IL-8 [17, 18, 22, 28].

The relationship between TNF-α, IL-8 expression, and MAPK signaling pathways in synovial fibroblasts has been studied using synovial fibroblasts from RA patients. However, to elucidate the mechanism of the onset of RA, it is necessary to use synovial fibroblasts without the occurrence of the inflammation response (including RA). Therefore, we investigated TNF-α-induced IL-8 protein expression and secretion and activation of MAPKs in canine synovial fibroblasts. Herein, we show that the distinctive activation of ERK and JNK MAPK signaling pathways contributes to TNF-α-induced IL-8 production and secretion. Moreover, we demonstrate that the specific isoforms of ERK and JNK, and ERK2 and JNK1, respectively, contribute to the effect of TNF-α.

## Material and methods

### Materials

A freezing vessel (BICELL) was obtained from Nihon Freezer Co., Ltd. (Tokyo, Japan). Dulbecco’s modified Eagle’s medium with 1 g/L glucose (DMEM-LG), phenylmethanesulfonyl fluoride (PMSF), sodium fluoride, and 4-(2-hydroxyethyl)-1-piperazineethanesulfonic acid (HEPES) were obtained from Wako Pure Chemical Industries, Ltd. (Osaka, Japan). TRIzol and Lipofectamine 2000 were purchased from Life Technologies Co. (Carlsbad, CA). Thermal Cycler Dice Real Time System II, TP900 DiceRealTime v4.02B, SYBR Premix Ex Taq II, PrimeScript RT Master Mix, and CELLBANKER 1 plus medium were purchased from TaKaRa Bio Inc. (Shiga, Japan). FR180204, SP600125, SB239063, SKF86002, anti-β-actin mouse monoclonal antibody (AC74), scramble siRNA, and siRNA for ERK1, ERK2, JNK1, and JNK2 were purchased from Sigma-Aldrich Inc. (St Louis, MO). Rabbit monoclonal antibodies against human total JNK-1 [t-JNK1, EPR140(2)] and human total JNK-2 (t-JNK2, EP1595Y) were obtained from Abcam (Cambridge, UK), and antibodies against rat total-ERK1/2 (t-ERK1/2, 137F5) and human phospho-ERK1/2 (p-ERK1/2, D13.14.4E) were obtained from Cell Signaling Technology Japan, K.K. (Tokyo, Japan). Rabbit polyclonal antibody against phospho-JNK (p-JNK) was obtained from Promega Co. (Madison, WI). Fluorescein isothiocyanate (FITC)-conjugated mouse monoclonal antibodies against human CD14, human CD29, human CD90, and human HLA-DR, FITC-conjugated rat monoclonal antibody against canine CD45, and phycoerythrin (PE)-conjugated mouse monoclonal antibodies against canine CD34 and canine CD44 were purchased from eBioscience Inc. (San Diego, CA). EC800 was purchased from Sony Co. (Tokyo, Japan). FLOWJO software was obtained from Tree star Inc. (Ashland, OR). ImageQuant LAS 4000 mini, horseradish peroxidase (HRP)-conjugated anti-mouse and anti-rabbit IgG antibodies, and ECL Western Blotting Analysis System were obtained from GE Healthcare (Piscataway, NJ). Mini-PROTEAN TGX gel, polyvinylidene difluoride (PVDF) membranes, and iCycler were purchased from Bio-Rad (Hercules, CA). Block Ace and Complete mini EDTA-free protease inhibitor mixture were obtained from Roche (Mannheim, Germany). An enzyme-linked immunosorbent assay (ELISA) kit for canine IL-8 was purchased from R&D Systems, Inc. (Minneapolis, MN). StatMate IV was obtained from ATMS (Tokyo, Japan).

### Cell culture

Canine synovial fibroblasts prepared from synovium of the stifle joint of male beagle dogs were kindly provided by Ms. Aki Ohmori, Teikyo University School of Medicine. The dissociated cells were maintained in static culture in DMEM-LG supplemented with 10% FBS, in an incubator with 5% CO_2_ and at 37°C. The medium was changed once a week. Cells were cryopreserved and thawed as previously described [29, 30]. Briefly, cells were harvested using 0.25% trypsin-EDTA till they reached 90–95% confluence and resuspended in CELLBANKER 1 plus medium at a density of 2 × 10^6^ cells/500 μL. The cell suspension (500 μL) was placed into each sterilized serum tube; the tubes were then placed in a freezing vessel (BICELL) and cryopreserved at −80°C. Before experiments, tubes were removed from the BICELL vessel and immersed in a water bath at 37°C. The thawed cell suspension was transferred into a centrifuge tube containing DMEM-LG with 10% FBS and centrifuged at 300 ×*g* for 3 min. The pellet was resuspended in DMEM-LG with 10% FBS and transferred into a 75-cm^2^ culture flask. Static culture was then performed under the same conditions as prior to cryopreservation. Cells were harvested using 0.25% trypsin-EDTA once they reached approximately 90% confluence, and the collected cells were seeded at a density of 1 × 10^6^/75-cm^2^ culture flask. Experiments were performed with canine synovial fibroblasts from the fourth passage. Each experiment was performed with cells derived from a single donor.

### Flow cytometry

Canine synovial fibroblasts were characterized by flow cytometry analysis based on a previous report, with slight modifications [29]. Cells were placed in 5 mL round-bottom tubes, at 3 × 10^5^ cells/tube, with PBS containing 0.5% FBS and incubated with antibodies (FITC-conjugated mouse monoclonal antibodies against human CD14, human CD29, human CD90 and human HLA-DR, FITC-conjugated rat monoclonal antibody against canine CD45, and PE-conjugated mouse monoclonal antibodies against canine CD34 and canine CD44) at 4°C for 30 min. An equal number of cells incubated with respective isotype control antibodies were used as control samples. Data were analyzed by recording 10,000 events on EC800 FLOWJO software.

### Real-time RT-PCR

Real-time RT-PCR was performed as previously reported [30]. Total RNA was extracted with TRIzol from cultured canine synovial fibroblasts, in accordance with the manufacturer’s instructions. Synthesis of cDNA was performed using PrimeScript RT Master Mix and carried out with 500 ng of total RNA. Real-time RT-PCR was performed with 2 μL of first-strand cDNA in 25 μL (total reaction volume), SYBR Premix Ex Taq II, and primers targeting canine IL-8 (forward: 5´-cacctcaagaacatccagagct-3´, reverse: 5´-caagcagaactgaactaccatcg-3´) or the TATA box binding protein (TBP, forward: 5´-ggcggatcaagtgttggaagggag-3´, reverse: 5´-acgcttgggattgtattcggcatta-3´), as the housekeeping gene. Real-time RT-PCRs of “no-template” controls were performed with 2 μL of RNase- and DNA-free water. Additionally, real-time PCRs of “no-reverse transcription” controls were performed with 2 μL of each RNA sample. PCR was performed using Thermal Cycler Dice Real Time System II with the following protocol: 1 cycle of denaturation at 95°C for 30 sec, 40 cycles of denaturation at 95°C for 5 sec, and annealing/extension at 60°C for 30 sec. Analyses of results were performed by the second derivative maximum method and the comparative cycle threshold (ΔΔ Ct) method, using real-time RT-PCR analysis software. The amplification of TBP from the same amount of cDNA was applied as an endogenous control, while cDNA amplification from canine synovial fibroblasts at time 0 was used as the calibration standard.

### Western blotting

Western blotting was performed as in previous reports [30, 31]. Cells were lysed with a lysis buffer containing 20 mM HEPES, 1 mM PMSF, 10 mM sodium fluoride, and a complete mini EDTA-free protease inhibitor cocktail, at pH 7.4. Protein concentrations were adjusted using the Bradford method [32]. Extracted proteins were boiled at 98°C for 5 min in SDS buffer, and samples were loaded into separate lanes of 12% Mini-PROTEAN TGX gel and electrophoretically separated. Separated proteins were transferred to PVDF membranes, treated with Block Ace for 50 min at room temperature, and incubated with primary antibodies [p-ERK1/2 (1:1,000), t-ERK1/2 (1:1,000), p-JNK (1:1,000), t-JNK1 (1:1,000), t-JNK2 (1:1,000), and β-actin (1:10,000)], for 120 min at room temperature. After washing, membranes were incubated with HRP-conjugated anti-rabbit or anti-mouse IgG (1:10,000), for 90 min at room temperature. Immunoreactivity was detected using ECL Western Blotting Analysis System. Chemiluminescent signals of membranes were measured using ImageQuant LAS 4000 mini.

### IL-8 ELISA

Canine synovial fibroblasts were seeded, at a density of 3.0 × 10^5^ cells/well, in 6-well culture plates. Cells starved for 24 h were treated with TNF-α for 0–24 h, and the culture medium was collected. IL-8 concentration in the culture medium was measured using an ELISA kit, according to the manufacturer’s instructions.

### siRNA transfection

siRNA transfection was performed as previously described, with slight modifications [30, 33]. Canine synovial fibroblasts, seeded at a density of 1 × 10^5^ cells/35 mm dish or 5 × 10^5^ cells/90 mm dish, were transfected using Opti-MEM containing 10 μL/mL Lipofectamine 2000 and 100 nM ERK1, ERK2, JNK1, JNK2, or scramble siRNA, for 6 h. After the transfection, the medium was changed to DMEM-LG containing 10% FBS, and the cultures were maintained in an incubator with 5% CO_2_ at 37°C for five days.

### Statistical analysis

Data from all experiments are presented as the mean ± standard error. Statistical analyses were performed using StatMate IV. Data from the time-course study were analyzed using two-way analysis of variance (ANOVA). Data from other experiments were analyzed using one-way ANOVA. Tukey’s test was used as post-hoc analysis. *P*-values inferior to 0.05 were considered statistically significant.

## Results

### Characterization of synovial fibroblasts using flow cytometry

**Fig 1:**
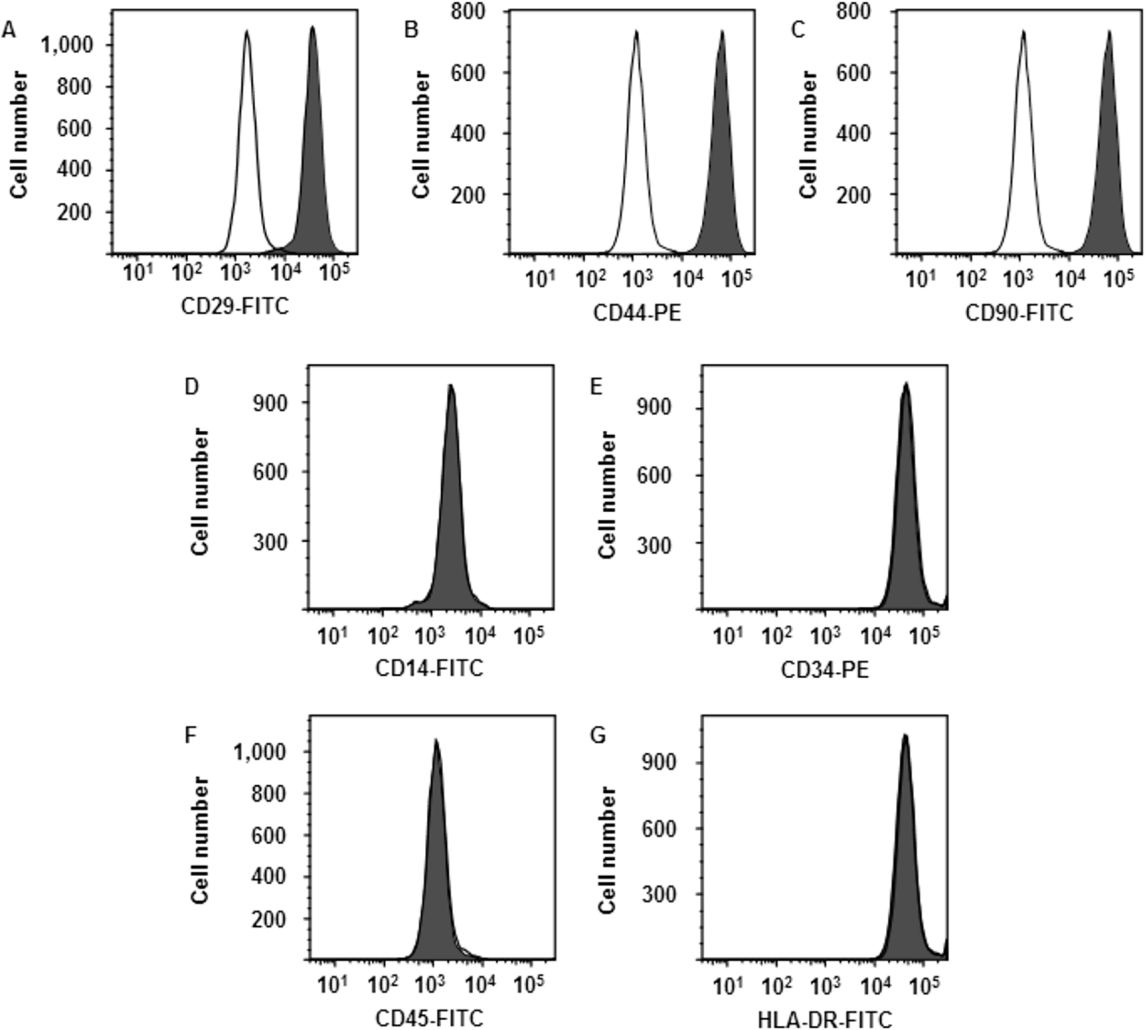
Characterization of synovial fibroblasts by flow cytometry. Synovial fibroblasts were isolated from three male beagle dogs. Solid and open histograms show non-specific and specific staining for the indicated marker, respectively. Cells were strongly positive for the fibroblast markers CD29, CD44, and CD90. In contrast, most of the cells were negative for the hematopoietic cell markers CD14, CD34, CD45, and HLA-DR. Results are representative in three independent experiments.

Flow cytometry was used to characterize canine synovial fibroblasts grown for four passages under control conditions. As shown in Fig 1, cells were strongly positive for the fibroblast markers CD29 (97.86 ± 1.23%), CD44 (97.40 ± 1.30%), and CD90 (97.50 ± 1.42%). In contrast, most of the cells were negative for the hematopoietic cell markers CD14 (1.60 ± 0.50%), CD34 (1.12 ± 0.10%), CD45 (0.97 ± 0.13%), and HLA-DR (2.73 ± 1.45%). These results are consistent with previous reports [34–36], indicating that the cells were synovial fibroblasts.

### TNF-α-induced IL-8 protein secretion and mRNA expression in synovial fibroblasts

**Fig 2:**
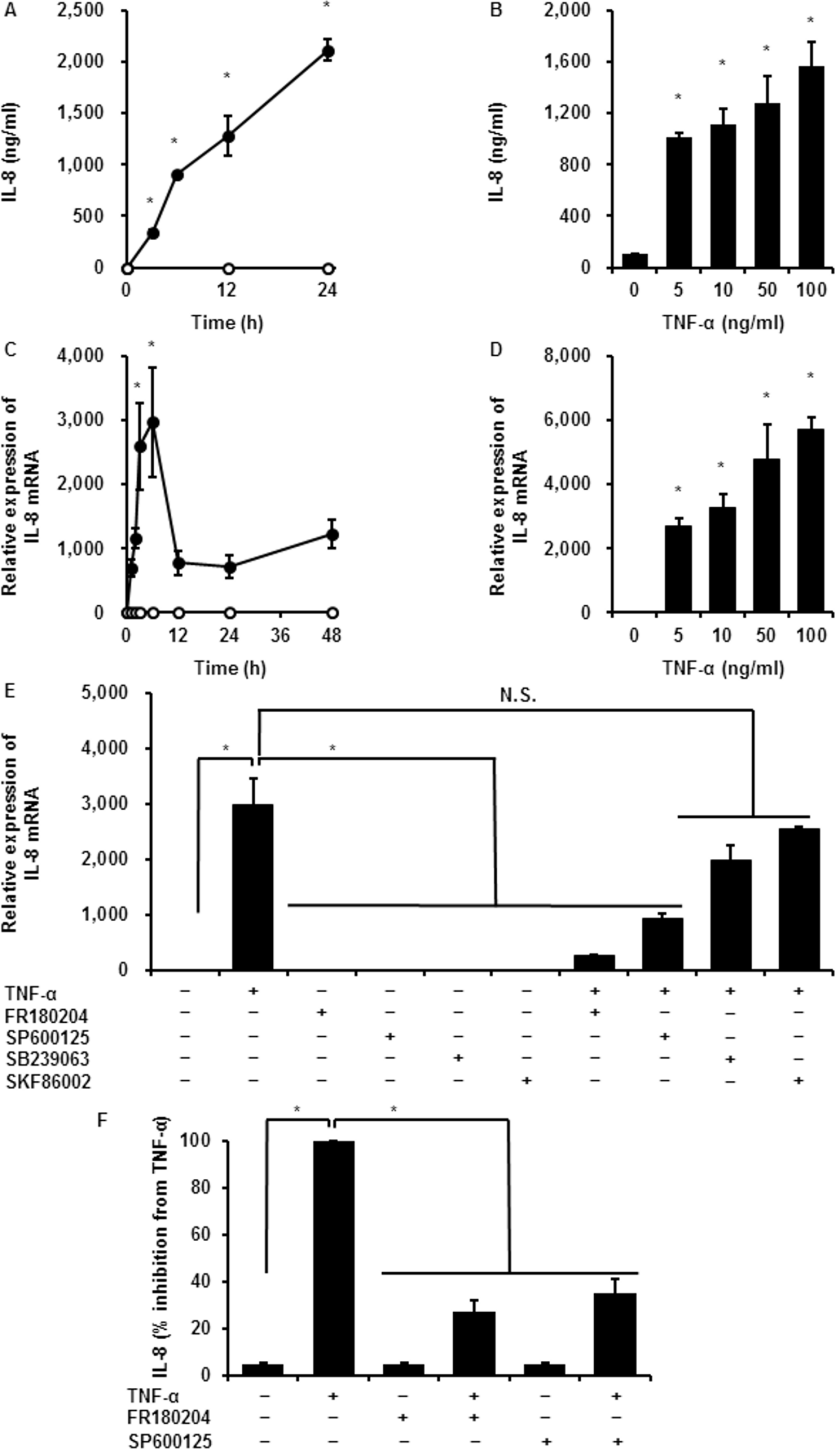
TNF-α-induced IL-8 protein secretion and mRNA expression in synovial fibroblasts. IL-8 protein secretion (A) and IL-8 mRNA expression (C) increased in a time-dependent manner in synovial fibroblasts incubated with (closed circle) or without (open circle) 100 ng/mL TNF-α, for the indicated time periods. IL-8 protein secretion (B) and mRNA expression (D) showed TNF-α dose-dependent stimulation. After pretreatment with the ERK1/2 inhibitor FR180204 (25 μM), the JNK1/2 inhibitor SP600125 (10 μM), and the p38 inhibitors SB239063 (20 μM) and SKF86002 (10 μM) for 1 h, synovial fibroblasts were stimulated with TNF-α (50 ng/mL) for 6 h. ERK1/2 and JNK1/2 inhibitors significantly attenuated TNF-α-induced IL-8 mRNA expression, whereas p38 inhibitors had no effect (E). ERK1/2 and JNK1/2 inhibitors also inhibited TNF-α-induced IL-8 protein secretion (F). Results are presented as mean ± SE from three independent experiments. Synovial fibroblasts isolated from three male beagle dogs were used, and each experiment was performed with cells derived from a single donor. *P < 0.05, compared with 0 h (A, C) or 0 pM (B, D).

TNF-α induces IL-8 secretion in various kinds of cells. Therefore, we first examined the effects of TNF-α on the secretion and expression of IL-8 in synovial fibroblasts by ELISA and real-time RT-PCR. When cells were treated with TNF-α (100 ng/mL) for 0–24 h, IL-8 concentration in the culture medium increased in a time-dependent manner (Fig 2A). Incubation with different doses of TNF-α (0–100 ng/mL) for 24 h led to a dose-dependent enhancement of IL-8 secretion (Fig 2B). Regarding the effect of TNF-α on IL-8 mRNA expression, TNF-α increased IL-8 mRNA expression in a time- and dose-dependent manner (Fig 2C and 2D). Taken together, the results suggest that TNF-α induces IL-8 protein secretion and mRNA expression in synovial fibroblasts.

### Involvement of MAPK signaling pathway in TNF-α-induced IL-8 mRNA expression

In mammalian cells, MAPK signaling pathway plays a pivotal role in several cellular responses, including inflammation. The expression of three major MAPKs (ERK, JNK, and p38 MAPK) and the detection of their active, phosphorylated forms have been demonstrated in synovial tissues and fibroblasts of RA patients [27]. Therefore, we examined the involvement of MAPK signaling pathways in TNF-α-induced IL-8 mRNA expression in synovial fibroblasts, using pharmacological inhibitors. In cells pretreated with the ERK1/2 inhibitor FR180204 (25 μM) or the JNK1/2 inhibitor SP600125 (10 μM), TNF-α failed to induce IL-8 mRNA expression, whereas TNF-α had less effect on IL-8 mRNA expression in the cells pretreated with the p38 inhibitors SB239063 (20 μM) and SKF86002 (10 μM) (Fig 2E). To confirm whether p38 signaling was activated, we examined the phosphorylation of p38 in TNF-α-treated cells. As shown in S1 Fig, TNF-α had no effect on the phosphorylation of p38. Next, we examined the effect of the ERK1/2 or JNK1/2 inhibitors on TNF-α-induced IL-8 secretion, observing that the inhibitors clearly attenuated TNF-α-induced IL-8 secretion (Fig 2F). These results suggest that TNF-α-induced IL-8 expression is predominantly dependent on the activation of ERK1/2 and JNK1/2 signaling pathways.

### Contribution of ERK isoforms to TNF-α-induced IL-8 mRNA expression

**Fig 3:**
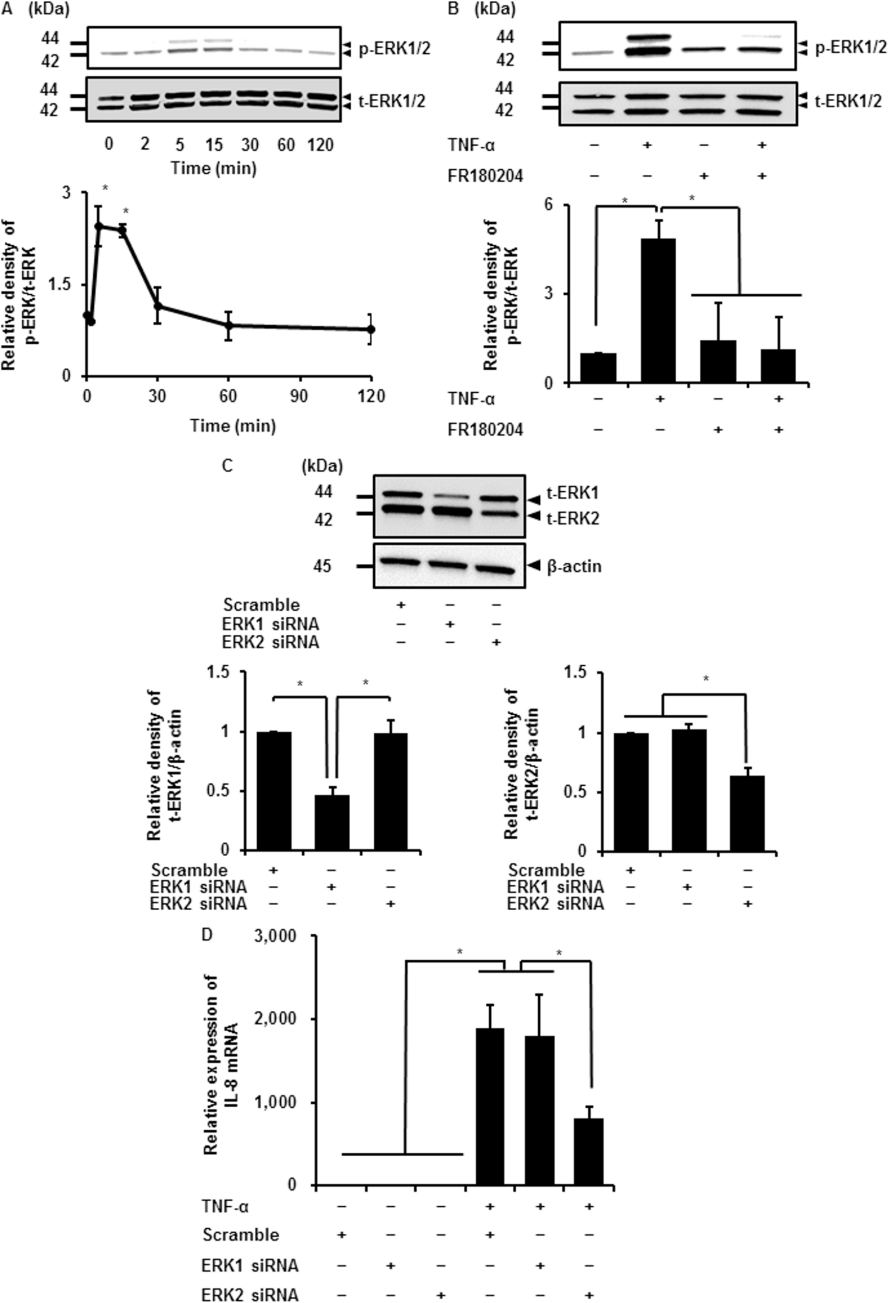
Contribution of ERK isoforms to TNF-α-induced IL-8 expression. (A) The levels of phosphorylated ERK1/2 (p-ERK1/2) and total ERK1/2 (t-ERK1/2) were detected by western blotting in cells treated with TNF-α (50 ng/mL) for 0–120 min (upper panel). Time-dependent change of relative densities of p-ERK1/2 compared with those at time 0 is shown (lower panel). (B) In cells pretreated with or without the ERK1/2 inhibitor FR180204 (25 μM) for 1 h and subsequently stimulated with or without TNF-α (50 ng/mL) for 5 min, TNF-α-induced ERK1/2 phosphorylation was clearly attenuated (upper panel). Relative density of attenuation of TNF-α-induced p-ERK compared with that in the absence of TNF-α is shown (lower panel). (C) ERK1 and ERK2 protein expression was significantly decreased in cells transfected with the respective siRNAs but not in scramble siRNA-transfected cells (upper panel). β-actin was used as an internal standard. Relative density of ERK1 and 2 in the respective siRNA-transfected cells compared with that in scramble siRNA-transfected cells is shown in lower left and lower right panels, respectively. (D) TNF-α-induced IL-8 mRNA expression was attenuated in cells transfected with ERK2 siRNA but not in those transfected with ERK1 or scramble siRNA. Cells transfected with ERK1, ERK2, or scramble siRNA were stimulated with or without TNF-α (50 ng/mL), for 6 h. Results are presented as mean ± SE from three independent experiments. Synovial fibroblasts isolated from three male beagle dogs were used, and each experiment was performed with cells derived from a single donor. *P < 0.05.

Given that ERK is active when in its phosphorylated form, we examined the effect of TNF-α on ERK1/2 phosphorylation (p-ERK1/2), by immunoblotting, using an anti-phospho-ERK1/2 antibody. When cells were stimulated with TNF-α (50 ng/mL), p-ERK1/2 increased in a time-dependent manner, reaching the maximum level after 5–15 min and then returning to the initial level (Fig 3A). TNF-α had no effect on total ERK1/2 (t-ERK1/2) expression. In cells pretreated with the ERK1/2 inhibitor FR180204, TNF-α failed to induce phosphorylation of ERK1/2, as shown in Fig 3B, suggesting that TNF-α activates ERK1/2 in synovial fibroblasts.

Two isoforms of ERK, ERK1 and 2, share similar biochemical properties and are co-expressed in almost all types of cells and tissues [37–40]. However, it is not clear whether ERK1 and 2 exert specific functions or act redundantly. To elucidate the contribution of ERK isoforms to the TNF-α-induced IL-8 expression, we performed an ERK1 or 2 knockdown experiment using ERK 1 or 2 siRNA transfection, respectively. As shown in Fig 3C, ERK1 and 2 protein expression was significantly decreased by the respective siRNAs but not by scramble siRNA transfection. TNF-α-induced IL-8 mRNA expression was attenuated in the ERK2 siRNA-transfected cells compared with the scramble or ERK1 siRNA-transfected cells (Fig 3D). These observations suggest that ERK2 predominantly contributes to the TNF-α-induced IL-8 expression in synovial fibroblasts.

### Contribution of JNK isoforms to TNF-α-induced IL-8 mRNA expression

**Fig 4:**
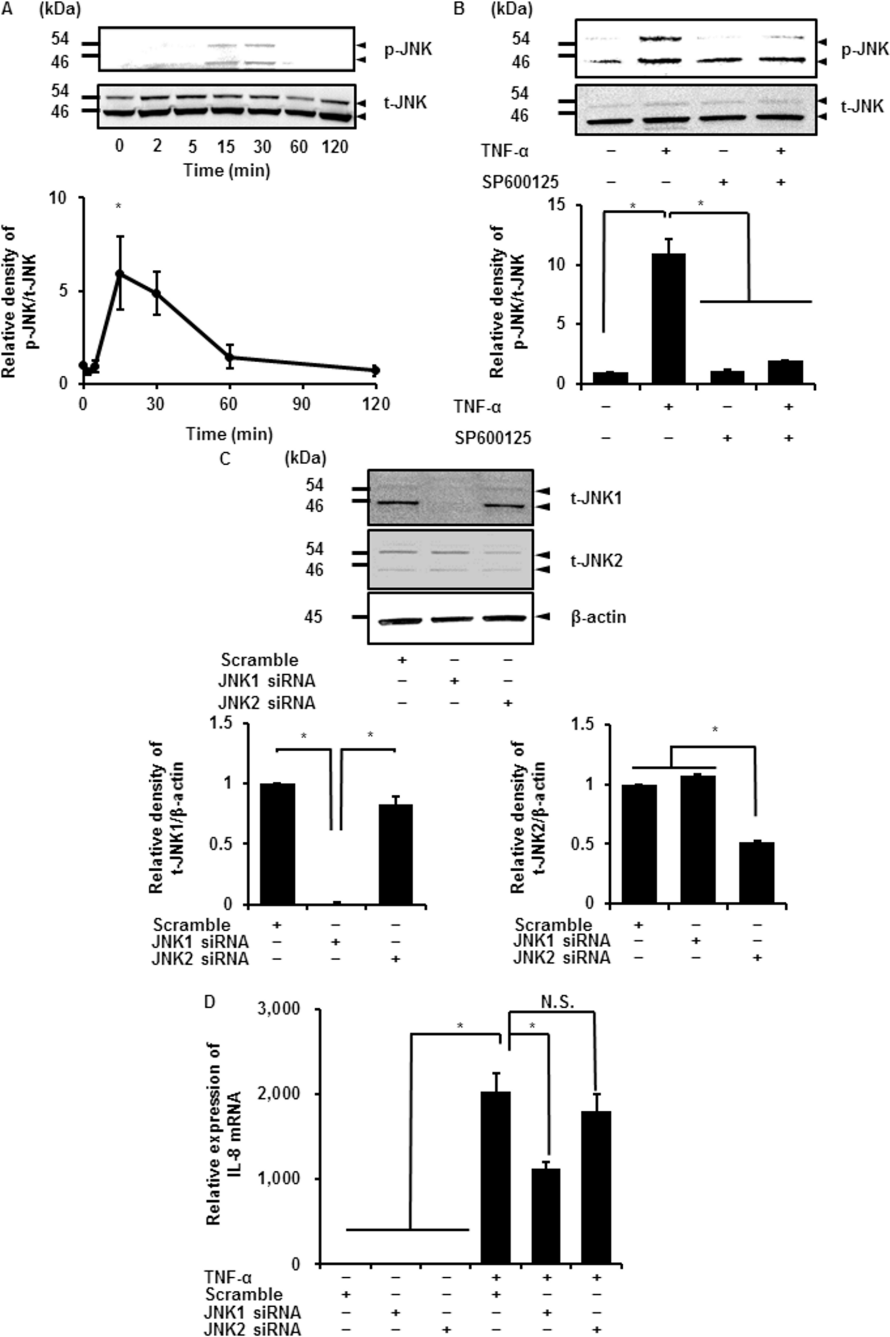
Contribution of JNK isoforms to TNF-α-induced IL-8 expression. (A) Expression of phosphorylated JNK1/2 (p-JNK1/2) and total JNK1/2 (t-JNK1/2) was detected by western blotting in cells treated with TNF-α (50 ng/mL) for 0–120 min (upper panel). Time-dependent change of relative density of p-JNK compared with that at time 0 is described (lower panel). (B) TNF-α-induced JNK1/2 phosphorylation was clearly attenuated in cells pretreated with the JNK1/2 inhibitor SP600125 (10 μM) for 1 h, and subsequently stimulated with TNF-α (50 ng/mL) for 15 min (upper panel). Relative density of the attenuation of TNF-α-induced p-JNK compared with that in the absence of TNF-α is shown (lower panel). (C) JNK1 and JNK2 protein expression was significantly decreased in cells transfected with the respective siRNAs, but not in scramble siRNA-transfected cells (upper panel). β-actin was used as an internal standard. Relative densities of JNK1 and 2 protein expression in the respective siRNA-transfected cells compared with that in scramble siRNA-transfected cells is shown in lower left and lower right panels, respectively. (D) TNF-α-induced IL-8 mRNA expression was attenuated in cells transfected with JNK1 siRNA but not in those transfected with JNK2 or scramble siRNA. The cells transfected with JNK1, JNK2, or scramble siRNA were stimulated with or without TNF-α (50 ng/mL) for 6 h. Results are presented as mean ± SE from three independent experiments. Synovial fibroblasts isolated from three male beagle dogs were used, and each experiment was performed with cells derived from a single donor. *P < 0.05.

JNK activation is mediated by phosphorylation, similarly to ERK1/2, and phosphorylates transcription factors, including c-Jun and activator protein-1 [41]. In synovial tissues of human RA patients, phosphorylation of JNK was elevated compared with levels in spondyloarthritis and undifferentiated arthritis patients [42]. TNF-α was previously reported to induce JNK phosphorylation in RA synovial fibroblasts, although changes in total JNK were unclear [43]. We investigated the effect of TNF-α on JNK1/2 phosphorylation. JNK1/2 phosphorylation (p-JNK1/2) occurred after TNF-α treatment in a time-dependent manner, reaching the maximum level after 15–30 min and then gradually returning to its initial level (Fig 4A), whereas no change in total JNK1/2 (t-JNK1/2) was observed (Fig 4A). In cells pretreated with the JNK inhibitor SP600125, TNF-α-induced JNK1/2 phosphorylation was clearly attenuated (Fig 4B). These observations suggest that TNF-α induces the phosphorylation of JNK1/2 in synovial fibroblasts.

Three isoforms of JNK, JNK1, 2, and 3, are encoded by three distinct genes. Of these, JNK1 and 2 are ubiquitously expressed, whereas JNK3 is expressed mainly in the heart, testes, and brain [44]. It is well known that isoforms JNK1 and 2 play a different role in regulating c-Jun expression and cell proliferation [45, 46]. To investigate the involvement of JNK1 and 2 in TNF-α-induced IL-8 expression, synovial fibroblasts were transfected with JNK1 or 2 siRNA. As shown in Fig 4C, JNK1 and 2 protein expression was significantly reduced by the respective siRNAs, but not by scramble siRNA transfection. In cells transfected with JNK1 siRNA, but not in those transfected with JNK2 siRNA, IL-8 mRNA expression induced by TNF-α was attenuated compared with the expression in cells transfected with the scramble siRNA (Fig 4D). To confirm whether JNK2 knockdown was effective for inactivation of JNK2 signaling, we examined the effect of JNK2 siRNA on TNF-α-induced mRNA expression of the other inflammatory cytokine interleukin 6 (IL-6). TNF-α-induced IL-6 mRNA expression was significantly reduced in the cells transfected with JNK2 siRNA, suggesting that JNK2 knockdown sufficiently attenuated the activation of TNF-α-induced JNK2 signaling (S2 Fig). Our results demonstrate that JNK1 plays a crucial role in the TNF-α-induced IL-8 expression in synovial fibroblasts.

### TNF-α induced distinctive activation of ERK2 and JNK1

**Fig 5:**
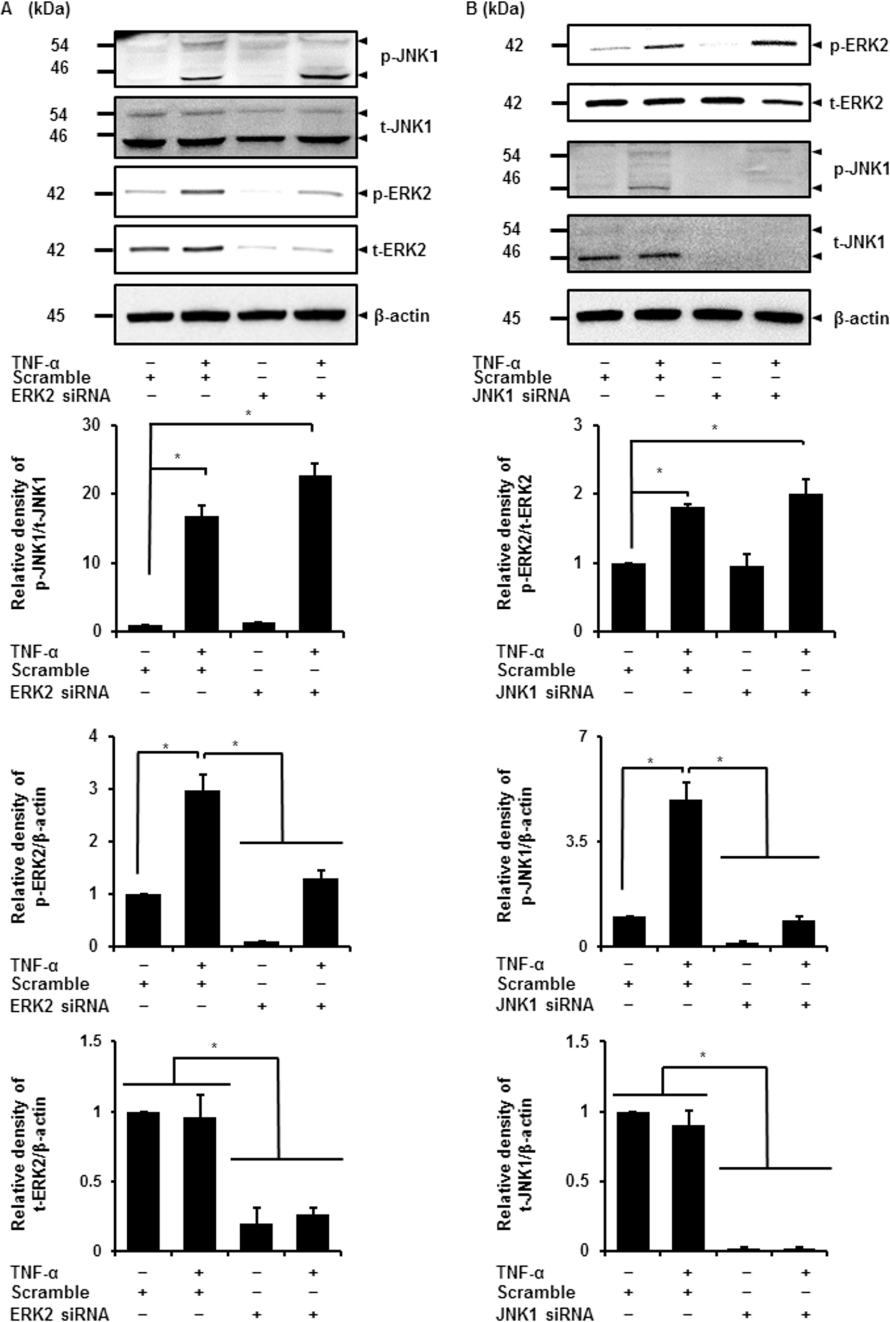
Parallel activation of ERK2 and JNK1 contributes to TNF-α-induced IL-8 expression. (A) Expression of phosphorylated JNK1 (p-JNK1), total JNK1 (t-JNK1), phosphorylated ERK2 (p-ERK2), total ERK2 (t-ERK2), and β-actin were detected by western blotting in cells transfected with ERK2 siRNA and subsequently stimulated with or without TNF-α (50 ng/mL) for 15 min (upper panel). β-actin was used as an internal standard. Relative densities of TNF-α-induced p-JNK1, p-ERK2, and t-ERK2 compared with those in the absence of TNF-α is shown in the lower panels. ERK2 siRNA transfection attenuated ERK2 protein expression and its phosphorylation, whereas no effect was observed on TNF-α-induced JNK1 phosphorylation. (B) Expression of phosphorylated ERK2 (p-ERK2), total ERK2 (t-ERK2), phosphorylated JNK1 (p-JNK1), total JNK1 (t-JNK1), and β-actin were detected by western blotting in cells transfected with JNK1 siRNA and subsequently stimulated with or without TNF-α (50 ng/mL) for 15 min (upper panel). β-actin was used as an internal standard. Relative densities of TNF-α-induced p-ERK2, p-JNK1, and t-JNK1 compared with those in the absence of TNF-α, are given in the lower panels. JNK1 siRNA transfection decreased the phosphorylation of JNK1, whereas no effect was observed on TNF-α-induced ERK2 phosphorylation. Results are presented as mean ± SE from three independent experiments. Synovial fibroblasts isolated from three male beagle dogs were used, and each experiment was performed with cells derived from a single donor. *P < 0.05.

Previous studies investigated the crosstalk between ERK and JNK signaling pathways. The ERK activator MEK was reported to regulate JNK activity in rat intestine cell line (IEC-6) and in human astrocyte cell line (U-251) [47, 48]. On the other hand, JNK activation was reported to inhibit ERK activation in COS-7 cells and in Bcr/Abl+ human leukemia cells [49, 50]. Recently, we demonstrated that IL-1β-induced JNK activation was necessary for MEK/ERK1/2 activation in feline synovial fibroblasts [33]. Therefore, herein, we investigated the interaction between ERK2 and JNK1 in TNF-α-treated synovial fibroblasts, using ERK2 and JNK1 siRNA transfection. In ERK2-knockdown cells, ERK2 protein expression and TNF-α-induced ERK2 phosphorylation levels were attenuated, but no effect on TNF-α-induced phosphorylation of JNK1 was observed (Fig 5A). In JNK1-knockdown cells, JNK1 protein expression and phosphorylation were attenuated, but TNF-α-induced ERK2 phosphorylation occurred (Fig 5B). We examined the effect of the co-transfection with ERK2 and JNK1 siRNAs on TNF-α-induced IL-8 mRNA. As shown in S3 Fig, TNF-α-induced IL-8 mRNA was reduced by the co-transfection, but the reduction level was no different from that by the single transfection with ERK2 or JNK1 siRNA, as mentioned above in Fig 3D and Fig 4D, respectively. These observations strongly suggest that ERK2 and JNK1 are distinctively activated by TNF-α.

## Discussion

In this study, we demonstrated that the pro-inflammatory cytokine TNF-α induced IL-8 mRNA expression and protein secretion in canine synovial fibroblasts. Therefore, it is conceivable that our experimental system is a useful model for the study of the mechanism of TNF-α-induced IL-8 expression in synovial fibroblasts.

Multiple MAPK signaling pathways are implicated in the regulation of key cellular processes, including arthritis and inflammation [51]. There are three major classes of MAPKs in mammals: p38 MAPK, ERK, and JNK. TNF-α stimulated secretion of IL-8 and, simultaneously, activated all three MAPKs in RA synovial fibroblasts [20, 22]. Since the effect of TNF-α on IL-8 secretion and MAPKs activation was inhibited by heat shock protein 70, the involvement of MAPKs in TNF-α-induced IL-8 secretion was suggested [22]. The contribution of p38 MAPK was also reported, as p38 inhibitors attenuated TNF-α-induced IL-8 in RA synovial fibroblasts [17, 19]. On the other hand, the inhibitors of all three MAPKs had no effect on TNF-α-induced IL-8 protein secretion in early passage RA synovial fibroblasts [20]. Furthermore, p38 inhibition was reported to enhance TNF-α-induced secretion of chemokines in RA synovial fibroblasts [52]. Such different results regarding the involvement of MAPKs in TNF-α-induced IL-8 expression appear to be due to synovial fibroblasts from RA patients with different conditions. When we used canine synovial fibroblasts as a model for the study, the involvement of ERK and JNK MAPK pathways, but not of p38, on TNF-α-induced IL-8 expression was observed. In normal human synovial fibroblasts, TNF-α was demonstrated to induce IL-8 secretion and to activate ERK and JNK, but not p38 MAPK [53]. Therefore, TNF-α-induced activation of p38 MAPK related to IL-8 expression may occur in synovial fibroblasts of RA patients.

In synovial fibroblasts from human RA patients, protein I/II and uric acid, a pathogen-associated molecular pattern from oral streptococci and a major component secreted from necrotic cells, respectively, have been demonstrated to stimulate IL-8 production and secretion via ERK1/2 and JNK [53, 54] Cyr61/CCN1, a product of an immediate early gene with roles in the mediation of cell adhesion and induction of cell migration, also stimulated IL-8 expression via ERK1/2 and JNK activation in RA synovial fibroblasts [55]. In human patients with RA developing progressive joint destruction, the enhancement of ERK1/2 and JNK activation was observed [42]. Together, these observations support that the activation of ERK1/2 and JNK are involved in IL-8 expression in synovial fibroblasts, which contributes to the pathogenesis of synovitis in RA, although the relationship between protein I/II, uric acid, or Cyr61 and TNF-α is unclear.

We also demonstrated that ERK2 and JNK1, isoforms of ERK and JNK, respectively, contribute to TNF-α-induced IL-8 expression in synovial fibroblasts. ERK exists in different isoforms, of which the most widely studied are ERK1 and ERK2. ERK1 and ERK2 share 83% amino acid identity and are co-expressed in most tissues [38, 56]. These two isoforms are generally co-activated in response to multiple extracellular stimuli [57–59]. In previous studies, the functional difference between the two isoforms was unclear, as activated alleles or isoform-specific inhibitors were unavailable [40, 60]. Currently, antisense techniques or siRNA transfection have been introduced as very specific ways to inhibit ERK isoforms in several types of cells, including synovial fibroblasts [33, 56, 61–66]. Therefore, we performed ERK-knockdown experiments by transfecting cells with ERK isoform-specific siRNA. In ERK2-knockdown cells, TNF-α-induced IL-8 mRNA expression was attenuated, but not in ERK1-knockdown cells, indicating that ERK2 is the dominant isoform in TNF-α-induced IL-8 expression in synovial fibroblasts.

Ten isoforms of JNK have been identified and are encoded by three distinct genes: JNK1, JNK2, and JNK3. While JNK1 and JNK2 are ubiquitously expressed in various tissues, JNK3 is mostly restricted to the heart, testes, and brain [44, 67]. Mice deficient in either JNK1 or JNK2 genes exhibit different phenotypes [40, 44]. In JNK1^−^/^−　^and JNK2^−^/^−^ mouse embryonic fibroblasts, the proliferation rate was differentially inhibited [68]. In this study, we demonstrated, by siRNA transfection, that JNK1, but not JNK2, contributes to the TNF-α-induced IL-8 expression in synovial fibroblasts. A JNK inhibitor suppressed joint destruction in rat adjuvant-induced arthritis [69], and a JNK1-inhibiting peptide abrogated synovial inflammation in mouse RA models (K/BxN) [70]. These observations support that JNK1 induces synovial inflammation and plays a crucial role in RA pathogenesis.

The interaction between JNK and MEK/ERK signaling has been reported in several types of cells. In rat intestine epithelial cells (IEC-6), the activation of ERK regulated JNK activation in camptothecin-induced apoptosis [48]. In human astrocyte cell lines (U-251) and *Xenopus laevis* oocytes, the positive feedback loop between Raf/MEK/ERK signaling and JNK signaling was reported [71]. On the other hand, the existence of a negative feedback loop between JNK and ERK signaling was also reported. In COS-7 cells, sustained JNK activation attenuated ERK activation [49]. In BCR/ABL+ human leukemia cells, the activation of JNK signaling was coordinated with ERK signaling in apoptosis induced by an histone deacetylase inhibitor [50]. In feline synovial fibroblasts stimulated with IL-1β, we demonstrated that JNK activation increased MEK/ERK phosphorylation [33]. However, in our study, ERK2 siRNA transfection had no effect on JNK1 phosphorylation, and JNK1 siRNA transfection also failed to inhibit ERK2 phosphorylation in TNF-α-stimulated synovial fibroblasts. These results suggest that activation of ERK2 and of JNK1 occurs independently in synovial fibroblasts stimulated with TNF-α. Since the balance between ERK1/2 and JNK activities after UV-C irradiation has been reported to control the cellular outcome in human fibroblasts [72], it is likely that different combinations of activation of MAPK isoforms control distinct intracellular signaling and cellular outcomes, which contributes to TNF-α-induced IL-8 expression in synovial inflammation of RA.

## Conclusions

**Fig 6:**
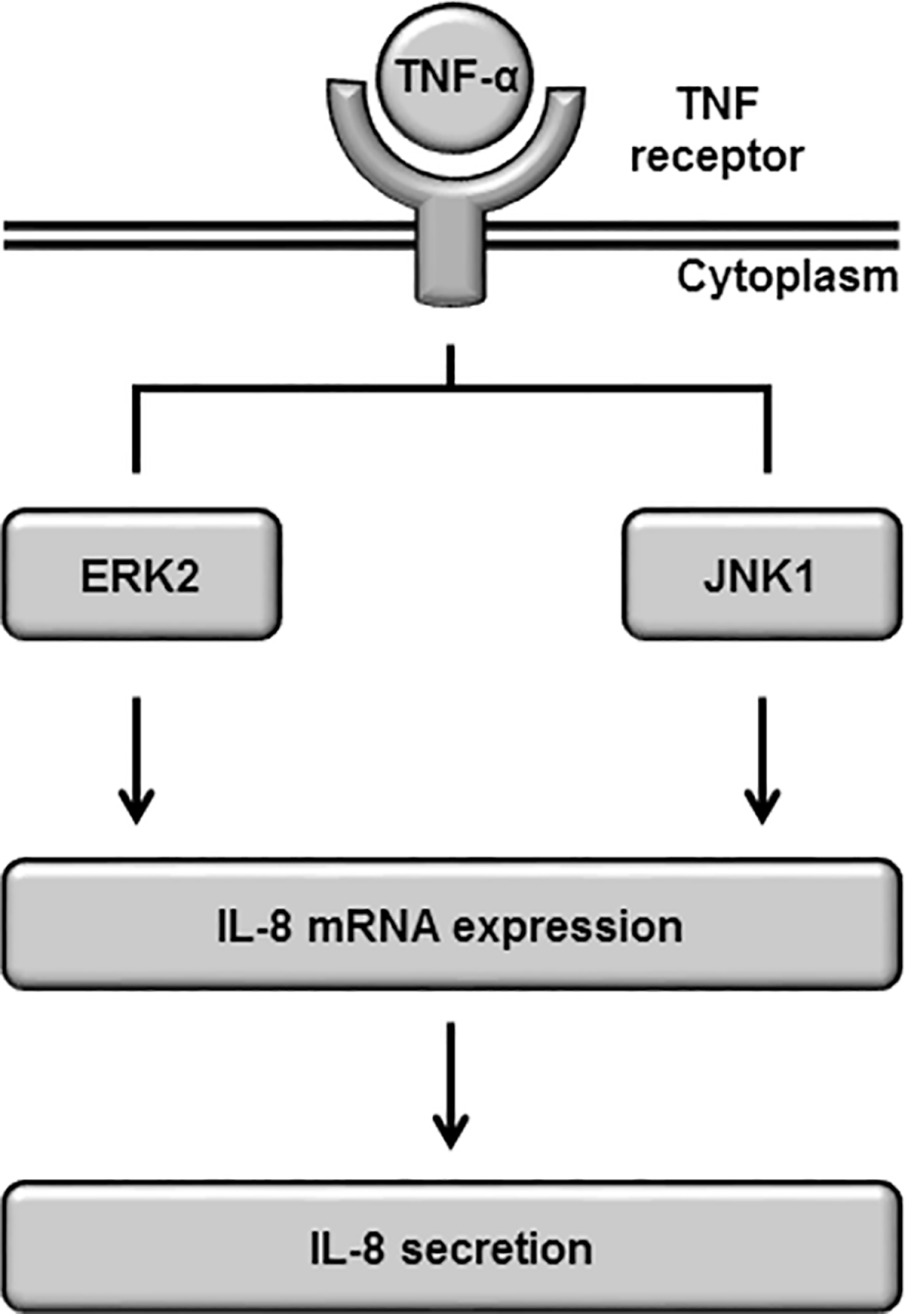
Schematic diagram of the contribution of ERK2 and JNK1 activation to TNF-α-induced IL-8 expression in synovial fibroblasts. In TNF-α-stimulated synovial fibroblasts, ERK2 and JNK1 cooperatively lead to the activation of IL-8 expression, without ERK1 and JNK2.

We demonstrated that the activation of ERK and JNK signaling contributes to TNF-α-induced IL-8 expression and secretion in synovial fibroblasts. Moreover, we demonstrated that the distinctive activation of specific MAPK isoforms, ERK2 and JNK1, is required for IL-8 expression. A scheme consistent with the summary in TNF-α-treated synovial fibroblasts is provided in Fig 6. Since MAPKs are considered potential therapeutic targets in RA [51]. our observations indicate that ERK2 and JNK1 represent promising molecular targets for therapeutic intervention in RA synovitis.

## Supporting information

S1 Fig No effect of TNF-α on p38 phosphorylation. (PDF)

S2 Fig Attenuation of TNF-α-induced expression of interleukin 6 (IL-6) mRNA in synovial fibroblasts transfected with JNK2 siRNA. (PDF)

S3 Fig The effect of ERK2 and JNK1 double knockdown on TNF-α-induced expression of IL-8 mRNA. (PDF)

S1 File Supplementary methods for real-time RT-PCR and western blotting. (PDF)
