# Supplementary material for: ERK2 and JNK1 contribute to TNF-α-induced IL-8 expression in synovial fibroblasts
Source: PLoS One. 2017 Aug 14;12(8):e0182923. doi: 10.1371/journal.pone.0182923 (PMC5555573; doi:10.1371/journal.pone.0182923)
Supplement: S1 Fig — (PDF) [file pone.0182923.s001.pdf]

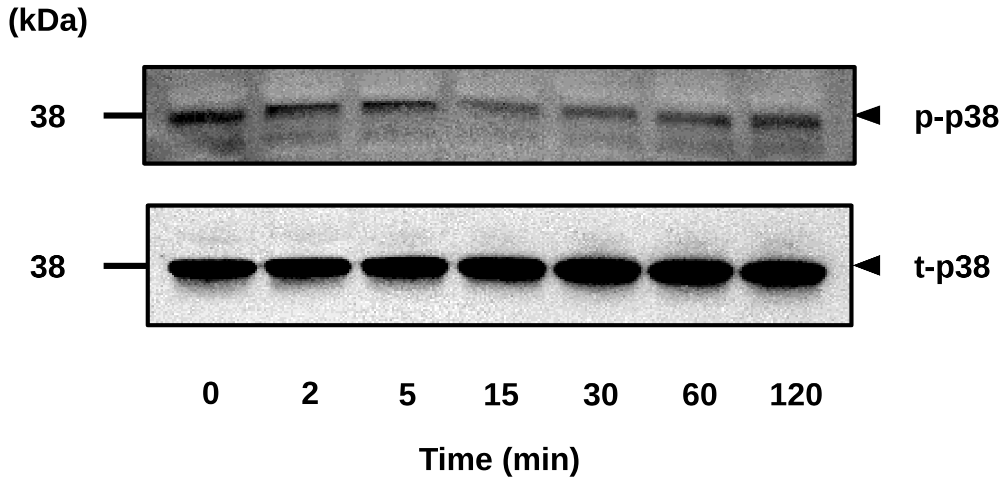

S1 Fig.
No effect of TNF-α on p38 phosphorylation. The levels of phosphorylated p38 (p-p38) and total p38 (t-p38) were detected by western blotting in synovial fibroblasts treated with 50 ng/mL TNF-α for 0-120 min. TNF-α failed to activate p38. Results are representative in three independent experiments. Synovial fibroblasts isolated from three male beagle dogs were used, and each experiment was performed with cells derived from a single donor.
