# Supplementary material for: ERK2 and JNK1 contribute to TNF-α-induced IL-8 expression in synovial fibroblasts
Source: PLoS One. 2017 Aug 14;12(8):e0182923. doi: 10.1371/journal.pone.0182923 (PMC5555573; doi:10.1371/journal.pone.0182923)
Supplement: S2 Fig — (PDF) [file pone.0182923.s002.pdf]

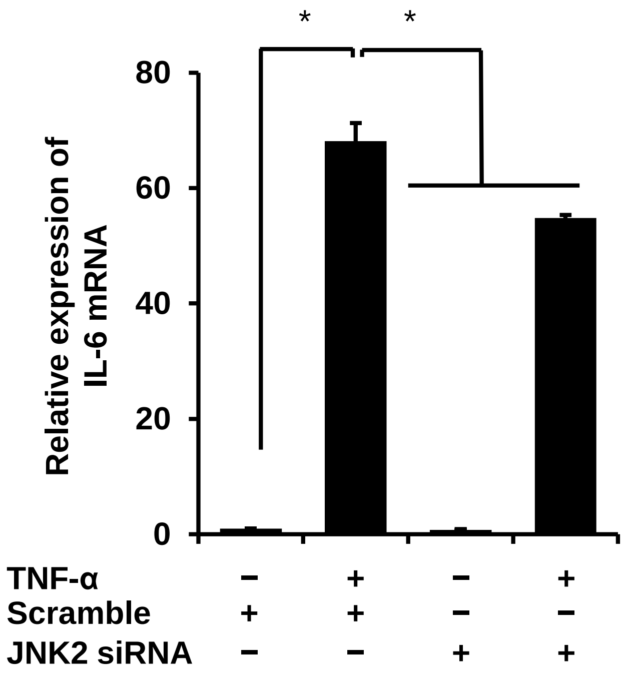

S2 Fig.

Attenuation of TNF-α-induced expression of interleukin 6 (IL-6) mRNA in synovial fibroblasts transfected with JNK2 siRNA. The cells transfected with JNK2 or scramble siRNA were stimulated with or without TNF-α (50 ng/mL) for 6 h. TNF-α-induced IL-6 mRNA expression was completely attenuated in cells transfected with JNK2 siRNA but not in those transfected with scramble siRNA. Results are presented as mean ± SE from three independent experiments. Synovial fibroblasts isolated from three male beagle dogs were used, and each experiment was performed with cells derived from a single donor. *$P < 0.05$. Data were analyzed using one-way ANOVA. Tukey's test was used as post-hoc analysis.
