# Supplementary material for: ERK2 and JNK1 contribute to TNF-α-induced IL-8 expression in synovial fibroblasts
Source: PLoS One. 2017 Aug 14;12(8):e0182923. doi: 10.1371/journal.pone.0182923 (PMC5555573; doi:10.1371/journal.pone.0182923)
Supplement: S3 Fig — (PDF) [file pone.0182923.s003.pdf]

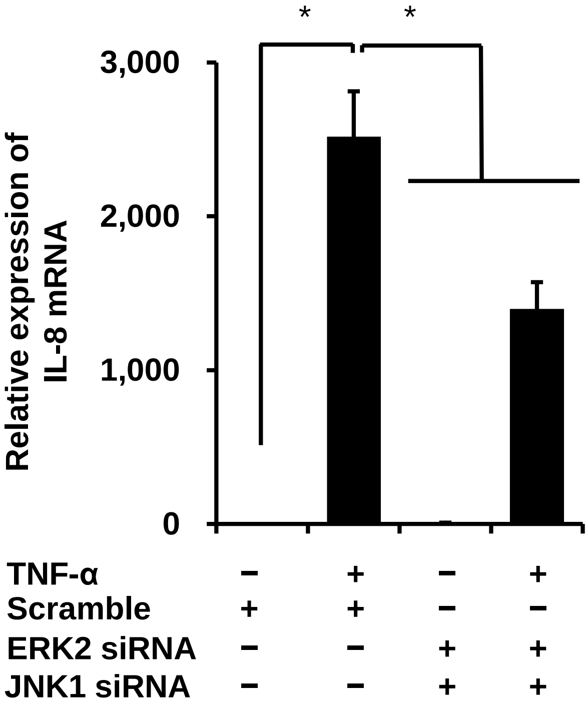

S3 Fig.

The effect of ERK2 and JNK1 double knockdown on TNF-α-induced expression of IL-8 mRNA. The synovial fibroblasts co-transfected with ERK2 and JNK1 siRNAs or transfected scramble siRNA were stimulated with or without TNF-α (50 ng/mL) for 6 h. TNF-α-induced IL-8 mRNA expression was partially decreased in cells co-transfected with ERK2 and JNK1 siRNAs but not in those transfected with scramble siRNA. Results are presented as mean ± SE from three independent experiments. Synovial fibroblasts isolated from three male beagle dogs were used, and each experiment was performed with cells derived from a single donor. *$P < 0.05$. Data were analyzed using one-way ANOVA. Tukey's test was used as post-hoc analysis.
