# Supplementary material for: ERK2 and JNK1 contribute to TNF-α-induced IL-8 expression in synovial fibroblasts
Source: PLoS One. 2017 Aug 14;12(8):e0182923. doi: 10.1371/journal.pone.0182923 (PMC5555573; doi:10.1371/journal.pone.0182923)
Supplement: S1 File — (PDF) [file pone.0182923.s004.pdf]

Supplementary Methods

Real-time RT-PCR

Total RNA was extracted with TRIzol from cultured canine synovial fibroblasts, in accordance with the manufacturer's instructions. Synthesis of cDNA was performed using PrimeScript RT Master Mix and carried out with 500 ng of total RNA. Real-time RT-PCR was performed with 2 μL of first-strand cDNA in 25 μL (total reaction volume), SYBR Premix Ex Taq II, and primers targeting canine IL-6 (forward: 5´-caagatcctggtccagatgctaaag-3´, reverse: 5´-cactcatcctgcgactgcaa-3´) or the TATA box binding protein (TBP, forward: 5´-ggcggatcaagtgttggaagggag-3´, reverse: 5´-acgcttgggattgtattcggcatta-3´), as the housekeeping gene. Real-time RT-PCRs of "no-template" controls were performed with 2 μL of RNase- and DNA-free water. Additionally, real-time PCRs of "no-reverse transcription" controls were performed with 2 μL of each RNA sample. PCR was performed using Thermal Cycler Dice Real Time System II with the following protocol: 1 cycle of denaturation at 95ºC for 30 sec, 40 cycles of denaturation at 95ºC for 5 sec, and annealing/extension at 60ºC for 30 sec. Analyses of results were performed by the second derivative maximum method and the comparative cycle threshold (ΔΔ Ct) method, using real-time RT-PCR analysis software. The amplification of TBP from the same amount of cDNA was applied as an endogenous control, while cDNA amplification from canine synovial fibroblasts at time 0 was used as the calibration standard.

Western blotting

Cells were lysed with a lysis buffer containing 20 mM HEPES, 1 mM PMSF, 10 mM sodium fluoride, and a complete mini EDTA-free protease inhibitor cocktail, at pH 7.4. Protein concentrations were adjusted using the Bradford method. Extracted proteins were boiled at 98ºC for 5 min in SDS buffer, and samples were loaded into separate lanes of 12% Mini-PROTEAN TGX gel and electrophoretically separated. Separated proteins were transferred to PVDF membranes, treated with Block Ace for 50 min at room temperature, and incubated with rabbit monoclonal antibodies against phosphorylated p38 (p-p38; 1:1,000, Cell Signaling Technology Japan, K.K.) or total p38 (t-p38; 1:1,000, Cell Signaling Technology Japan, K.K.) for 120 min at room temperature. After washing, membranes were incubated with HRP-conjugated anti-rabbit (1:10,000), for 90 min at room temperature. Immunoreactivity was detected using ECL Western Blotting Analysis System. Chemiluminescent signals of membranes were measured using ImageQuant LAS 4000 mini.
